# Label extension, single-arm, phase III study shows efficacy and safety of stempeucel® in patients with critical limb ischemia due to atherosclerotic peripheral arterial disease

**DOI:** 10.1186/s13287-023-03292-w

**Published:** 2023-04-01

**Authors:** Pawan Kumar Gupta, P. Shivashankar, M. Rajkumar, Subhendu S. Mahapatra, Sanjay C. Desai, Anita Dhar, Vinay Krishna, N. S. Raviraja, Samatha Bhat, Pachaiyappan Viswanathan, Suresh Kannan, Jijy Abraham, Hema Boggarapu, M. S. Manjuprasad, K. Udaykumar

**Affiliations:** 1 Stempeutics Research Pvt. Ltd., 3rd Floor, Manipal Hospitals Whitefield #143, EPIP Industrial Area, ITPL Main Road, Bangalore, 560 048 India; 2 Vijaya Hospital, Chennai, India; 3 Health Point Hospital, Kolkata, India; 4 M. S. Ramaiah Medical College & Hospitals, Bangalore, India; 5 All India Institute of Medical Sciences, New Delhi, India; 6 GSVM Medical College, Kanpur, India

**Keywords:** Mesenchymal stromal cells, Peripheral arterial disease, Ulcer healing, Ankle–brachial pressure index, Neovascularization

## Abstract

**Background:**

Peripheral arterial disease (PAD) of lower extremities comprises a clinical spectrum that extends from asymptomatic patients to critical limb ischemia (CLI) patients. 10% to 40% of the patients are at the risk of primary amputation. This study was planned in “no-option” patients of CLI due to atherosclerotic PAD to assess the efficacy and safety of pooled, allogeneic, adult human bone marrow-derived mesenchymal stromal cells which is already approved for marketing in India for CLI due to Buerger’s disease.

**Methods:**

This was a single-arm, multi-centric, phase III study where mesenchymal stromal cells was injected as 2 million cells/kg body weight in the calf muscle and around the ulcer. Twenty-four patients of lower extremity CLI due to PAD with Rutherford III-5 or III-6 and ankle–brachial pressure index ≤ 0.6 and having have at least one ulcer with area between 0.5 and 10 cm^2^ were included in the study. These patients were evaluated over 12 months from drug administration.

**Results:**

Over a period of 12 months, statistical significant reduction of rest pain and ulcer size along with improvement in ankle–brachial pressure index and ankle systolic was observed. The quality of life of patients improved together with increase in total walking distance and major amputation-free survival time.

**Conclusion:**

Mesenchymal stromal cells may be a feasible option to treat “no-option” patients with atherosclerotic PAD.

*Trial registration* This study is registered prospectively in National Institutes of Health and Clinical Trials Registry—India (CTRI) website: CTRI/2018/06/014436. Registered 6th June 2018. http://ctri.nic.in/Clinicaltrials/pmaindet2.php?trialid=24050&EncHid=&userName=stempeutics.

## Background

Peripheral arterial disease (PAD) is characterized by narrowing and blockade of upper and/or lower extremities, usually based on underlying obliterating atherosclerosis which reduces arterial flow [1, 2]. The PAD of lower extremities comprises a clinical spectrum that extends from asymptomatic patients to patients with critical limb ischemia (CLI) that might result in amputation and limb loss [3]. It is estimated that > 230 million people have PAD worldwide which is increasingly recognized as an important cause of cardiovascular morbidity and mortality [4]. More than 50% of PAD patients are asymptomatic and 20–30% of individuals have diabetes mellitus. Atherosclerosis accounts for more than 90% of cases and the prevalence of amputation is 3–4% in PAD patients [5]. Narrowed vessels due to spasm or built up of plaque that cannot supply sufficient blood flow to peripheral leg muscles cause claudication, which is brought on by exercise and relieved by rest [6]. Ischemic rest pain which is an intractable, burning pain in the soles of the feet results due to poor perfusion to the nerves and poor blood flow results in non-healing wounds and ischemic ulcers [7]. 10% to 40% of the CLI patients are at the risk of primary amputation [8].

The last available option for PAD patients who have exhausted their pharmacological or non-pharmacological approach and surgical option is amputation [9, 10]. It is estimated that the mortality rate in these patients who are not eligible for surgical revascularization or endovascular treatment, designated as “no-option” CLI patients, within 6 months from diagnosis is approximately 20%, and at 1 year it advances to 40%, while another 40% would undergo major limb amputation [11, 12].

As per clinical studies, stem cell-based therapy is feasible and may have beneficial effects when it is delivered to the ischemic muscle which has demonstrated therapeutic angiogenesis for treating ischemic tissue and preventing major amputation in CLI patients unsuitable for revascularization [13, 14]. The migratory capacity of stem cells is dependent on natural growth factors such as vascular endothelial growth factor (VEGF), stromal cell-derived factor 1 (SDF-1) and stem cell factor (SCF). The expression of VEGF, SDF-1 and SCF is highly unregulated in the hypoxic muscular tissue and is responsible for the recruitment of the stem cells to assist in the repair mechanism. These features enable the stem cells to promote post-ischemic neovascularization and blood flow recovery in ischemic diseases secondary to PAD [15].

Our previous phase II and phase IV study showed that the use of stempeucel® was safe and efficacious at a dose of 2 million cells/kg body weight in patients with CLI due to Buerger’s disease (BD) [16, 17]. This present study is planned to extend the label of the drug to CLI due to atherosclerotic PAD as the mechanism of action of the drug and the dose to be administered is similar that is 2 million cells/kg body weight.

## Methods

### Preparation and release criteria of stempeucel®

The methodology of isolating bone marrow, preparation of master cells bank, working cell bank and preparation of stempeucel® has been published previously and has been patented (US20110229965) [17]. Further the stability of the stempeucel® product (150 million and 200 million) was analyzed for 36 months and 24 months, respectively, as per International Council for Harmonization (ICH) guidelines. Stempeucel® was assessed for identity, purity, impurity, potency, sterility, safety and genetic stability against the stringent in-house specification before releasing for the study. The potency of stempeucel® which has the direct role in clinical significance was evaluated via critical validated test parameters—viable cell count, viability and VEGF potency assay [17].

### Study design

The product stempeucel® is already approved by Indian FDA for indication CLI due to BD. Hence, the present study was planned as a label extension, single-arm, multi-centric, phase III study to assess the efficacy and safety of stempeucel® in patients with CLI due to atherosclerotic PAD in Indian population. The protocol and informed consent were approved by the Indian FDA and institutional ethics committees of all 7 participating sites, and all subjects gave informed consent. The study was conducted as per International Council for Harmonization Good Clinical Practice (ICH-GCP) guidelines, principles of Declaration of Helsinki, Schedule Y of Drugs and Cosmetic Act, 1945, and Ethical guidelines for biomedical research on human participants, Indian Council of Medical Research (ICMR) 2006 and registered prospectively in Clinical Trials Registry—India (CTRI/2018/06/014436).

A total of 24 patients with unilateral lower extremity CLI due to PAD were enrolled into the study. All patients were injected with stempeucel® based on body weight (2 million viable cells/kg body weight) at baseline visit by a qualified physician as 0.6 ml/kg (if 200 million cells bag is used) or 0.8 ml/kg (if 150 million cells bag is used) of the reconstituted product at 40–60 multiple intramuscular injections in the gastrocnemius muscle in a volume of 0.5 or 1 ml per injection, depending on patient weight. Further, 8 million viable cells which are equivalent to 2 or 3 ml (for 200 or 150 million cell bags, respectively) were administered around the ulcer as multiple intramuscular/intradermal/subcutaneous injections based on the location of the ulcer. To decrease the risk of hypersensitivity reaction premedication of injection of 100 mg of hydrocortisone and 45.5 mg of pheniramine maleate was administered within 1 h prior to study drug injection to all patients.

#### Method of calculating dose

The viability of cells is presumed to be ≥ 85%. Based on this, the actual number of viable cells considered in 200 million cells bag and 150 million cells bag is 170 million cells and 128 million cells, respectively. Hence, the volume of the drug to be administered with a cell concentration of 2 million cells/kg is 0.8 ml/kg or 0.6 ml/kg for 150 million or 200 million cell bags, respectively.

#### Selection of patients and follow-up

The detailed inclusion/exclusion criteria of the patients are given in Table 1. The patients were followed regularly after initial diagnosis and evaluation at one week (Visit 3), one month (Visit 4), 3 months (Visit 5), 6 months (Visit 6) and 12 months (Visit 7) after administration of stempeucel® for efficacy and safety.

**Table 1:** Inclusion and exclusion criteria

*Inclusion Criteria*
1. Males or females with non-child bearing potential in the age group of 18–70 years of Indian origin
2. Established CLI, clinically and hemodynamically confirmed as per III-5 or III-6;
3. Patients in Rutherford III-6 if gangrene extending maximally up to the head of metatarsal but limited to toes (Patients with wet gangrene must undergo wound debridement/amputation before screening)
4. Patients having Infra-inguinal arterial occlusive disease (as evidenced by MRA) with rest pain or ischemic ulcer/necrosis, who are not eligible for or have failed traditional revascularization treatment (No option patients)
5. Patients should have at least one ulcer (target ulcer): area between 0.5 and 10 cm2 (both inclusive)
6. Ankle–brachial pressure index (ABPI) ≤ 0.6
7. Patients if having associated Type II Diabetes, should be on medication and well controlled (HbA1c ≤ 8%) without complications
8. Normal liver and renal function
9. On regular medication for hypertension if any
10. Patients who are able to understand the requirements of the study, and willing to provide voluntary written informed consent including audio – video consent, abide by the study requirements, and agree to return for required follow-up visits
*Exclusion Criteria*
1. Patients with CLI suitable for surgical or percutaneous revascularization as determined by the surgeon performing vascular procedure
2. Buerger’s Disease as diagnosed by Shionoya criteria
3. Patients with rest pain VAS score < 3 on rest pain scale of 0 – 10 (0 is no pain; 10 is maximum pain)
4. Ulcers with exposure of tendon and/bone in the shin region
5. Severe, active infection of the involved extremity, including osteomyelitis, fasciitis or severe/purulent cellulitis
6. CLI patient requiring amputation proximal to trans-metatarsal level
7. Patients with gait disturbance for reasons other than CLI
8. Type I diabetes
9. Patients having respiratory complications/left ventricular ejection fraction < 35%
10. Stroke or myocardial infarction within last 3 months
11. Patients with deep vein thrombosis in any limb
12. Patients who are contraindicated for MRA
13. Have clinically serious and/or unstable inter-current infection, medical illnesses or conditions that are uncontrolled or whose control, in the opinion of the investigator, may be jeopardized by participation in this study or by the complications of this therapy
14. Documented terminal illness or cancer or any concomitant disease process with a life expectancy of less than 1 year
15. Patients already enrolled in another investigational drug trial or completed within 3 months
16. Patients who have participated in any stem cell trial/therapy/gene therapy any time in the past
17. History of severe alcohol or drug abuse within 3 months of screening
18. Hb% < 10 gm% for males, Hb% < 9 gm% for females, serum creatinine ≥ 2 mg%, serum Total Bilirubin ≥ 2 mg%
19. Women with child bearing potential, pregnant and lactating women
20. Patient with known hypersensitivity to the constituents of the IMP– DMSO
21. Patients tested positive for HIV (1 or 2), HCV, HBV, CMV, TPHA
22. Subject is an employee or relative of any member of the Investigational site or the Sponsor
23. Refusal or inability to give informed consent including audio–video consent

CLI: Critical Limb Ischemia; MRA: Magnetic Resonance Angiography; ABPI: Ankle–Brachial Pressure Index; VAS: Visual Analogue Scale; IMP: Investigational Medicinal Product; DMSO: Dimethyl Sulfoxide; HIV: Human Immunodeficiency Virus; HCV: Hepatitis C Virus; CMV: Cytomegalovirus; TPHA: Treponema Pallidum Hemagglutination Assay

#### Efficacy evaluation

The primary efficacy assessment was the relief of rest pain and the healing of ulcerations in the target limb. Complete ulcer healing was defined as complete epithelization of ulcer (100% reduction in ulcer size from the baseline assessment) and partial ulcer healing as at least 30% decrease in ulcer size to the baseline assessment. Ulcer size in cm^2^ was measured by standardized measurement using WoundZoom (digital, non-contact wound documentation camera system).

The secondary efficacy assessment included improvement in ankle–brachial blood pressure (ABPI) and ankle systolic pressure (ASP) measured by Doppler, improvement in total walking distance (TWD) as measured by treadmill, improvement in quality of life (QoL) as measured by the King’s College VascuQOL questionnaire and major amputation-free survival.

#### Safety evaluation

Safety assessment included evaluation of adverse effects (AEs), treatment emergent AEs (TEAEs) vital signs, physical examination and occurrence of clinical abnormality at site of injection, calf area and clinical laboratory parameters, physical examinations and electrocardiogram.

### Statistical analysis

#### Sample size calculation

Sample size for the study was based on the inputs from data of change in rest pain and ulcer healing from baseline to 6-month follow-up in phase II study in CLI due to BD [13]. A sample size of 24 patients was required to achieve a 90% power to denote the change in rest pain and ulcer size from baseline to 6 months with effect size of 3.19 and 1.23 and assuming a standard deviation (SD) of 1.6 and 0.49, respectively, an alpha value of 0.05 (two-sided test) and an expected dropout rate of 20%.

#### Analysis of data

The statistical analysis to assess the efficacy and safety end points was carried out using SAS® Version 9.4 (SAS Institute Inc., USA). Data are presented as mean ± SD. Normality of continuous data was tested using Shapiro–Wilk test. Change in rest pain, ulcer size, ABPI, ASP, improvement in TWD and QoL from baseline to each visit were evaluated using Wilcoxon sign rank test or paired t test based on normality of the data. The efficacy parameters were also analyzed by using a generalized estimating equation (GEE) model with longitudinal analysis and chi-square test as appropriate. A *p* value < 0.05 was considered to indicate a statistically significant difference.

## Results

### Potency and stability of the drug product

**Fig. 1:**
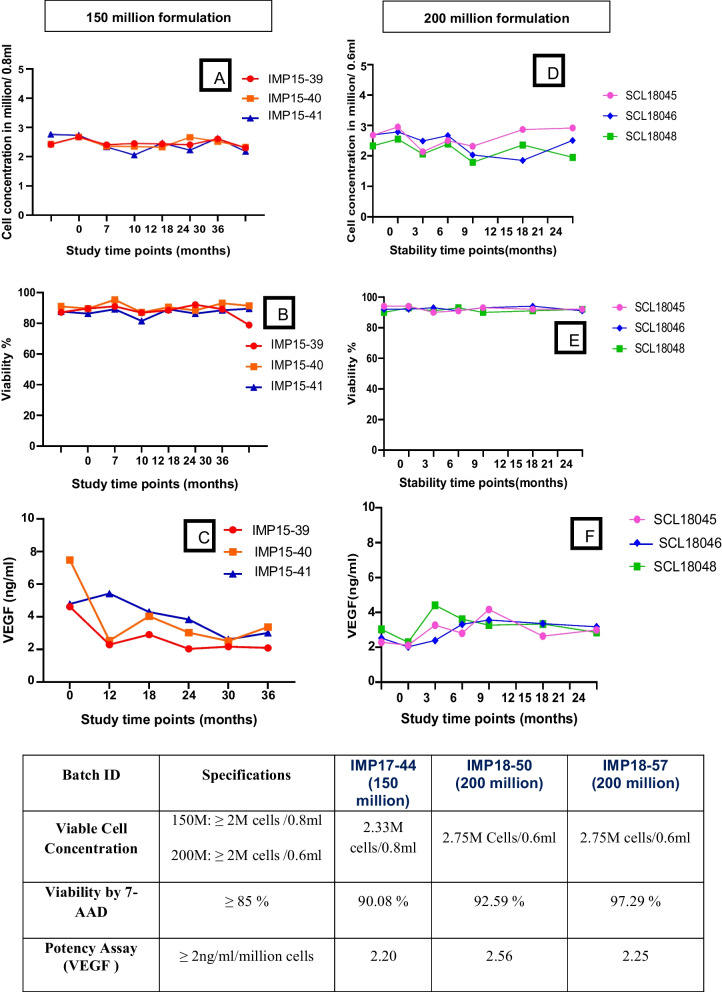
Stability of Stempeucel 150 M and 200 M. Preparations 150 M (**A**, **B** and **C**) and 200 M (**D**, **E** and **F**) were analyzed for their potency using viable cell concentration, viability and potency assay at various time points as per International Council for Harmonization guidelines

One batch of stempeucel® 150 million and two batches of 200 million was manufactured, tested and released for study. The 150 M batch was stable for 36 months, and 200 M batch was stable for 24 months. The viable cell count was 2.33 million cells per 0.8 ml (between 2.18 and 2.76 million at various time points) in 150 M batch and 2.75 million cells per 0.6 ml (between 1.8 and 2.96 million cells at various time points) in both 200 M batches. The minimum limit of cell concentration as per in-house criteria was ≥ 2 million cells. Viable cell count of all the three batches was much above the limit of recommended clinical dose. The identity, purity, potency, genetic stability, safety and impurity of stempeucel® were assessed in all the three batches qualified as per in-house established specification (Fig. 1).

**Fig. 2:**
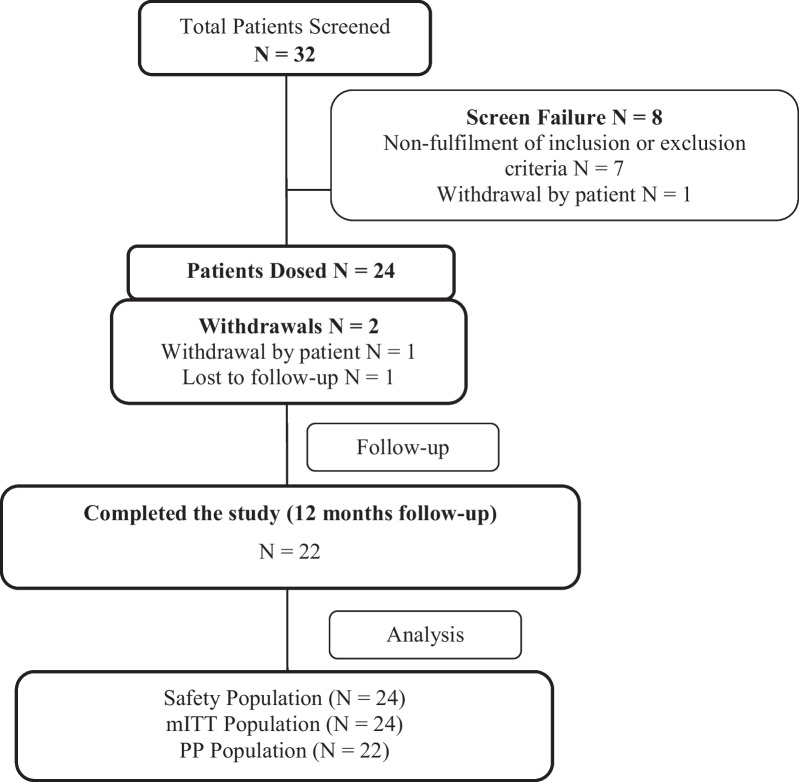
CONSORT diagram showing number of patients enrolled, followed up and analyzed

After screening 32 patients, 24 (21 males and 3 females) of them were enrolled and dosed (included under mITT population) having a mean age of 53 years and weight 60.8 kg (Table 2). The CONSORT diagram (Fig. 2) shows the number of patients screened, enrolled and completed the 12-month follow-up.

**Table 2:** Demography and baseline characteristics of patients

Parameter (Units)	Safety Population (N = 24)
Age (years), Mean ± SD	53 ± 8.3
Weight (kg), Mean ± SD	60.8 ± 11.99
Sex (Male), n (%)	21 (87.5%)
Sex (Female), n (%)	3 (12.5%)
Height (cms), Mean ± SD	162.4 ± 7.98
N: number; SD: Standard Deviation

## Efficacy results (Fig. 3)

**Fig. 3:**
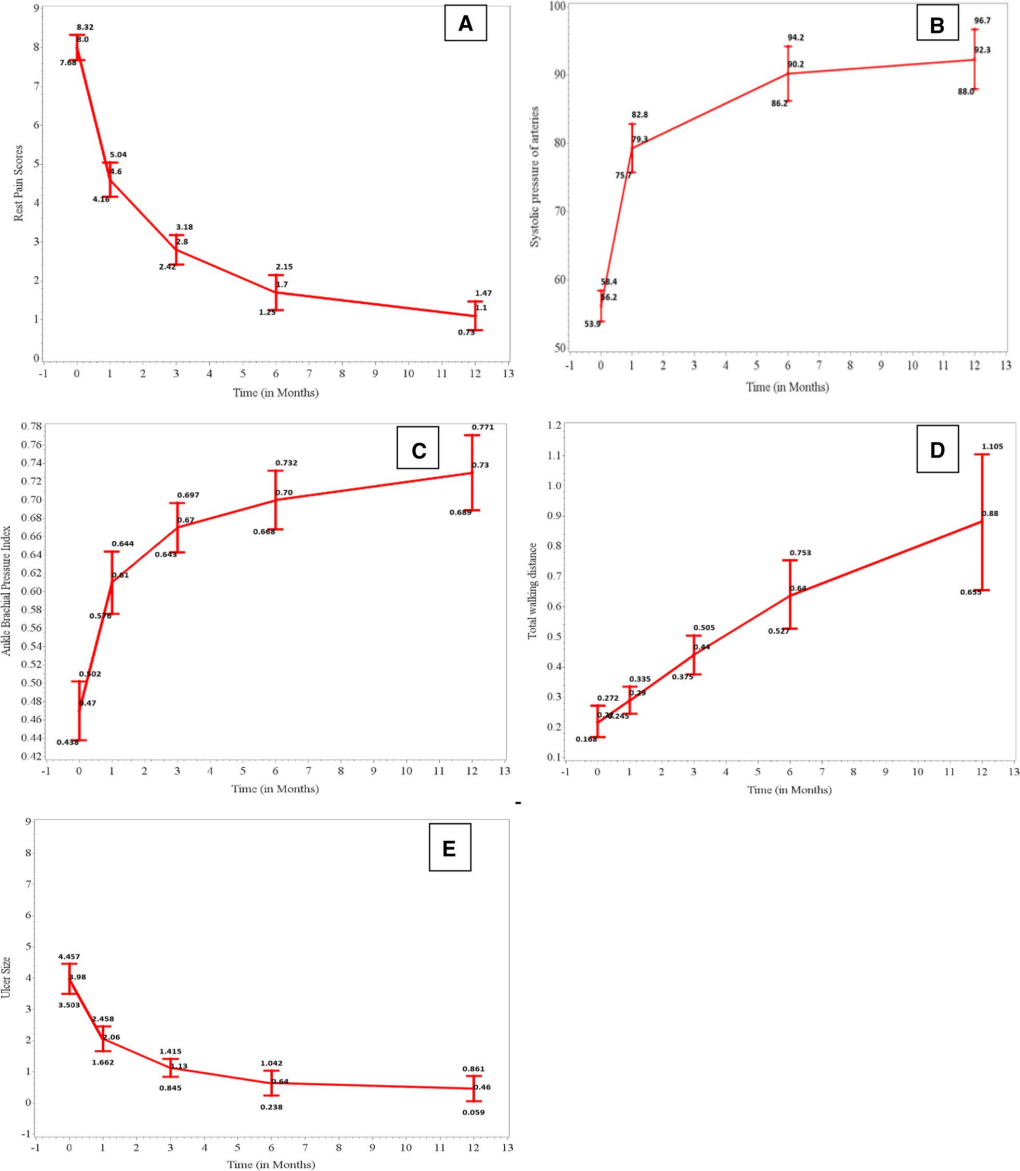
Change in efficacy parameters over time compared to baseline. **A** Rest pain score over 12 months improved compared to baseline (*P* value 0.0001), **B** Ankle systolic pressure over 12 months improved compared to baseline (*P* value 0.0001), **C** Ankle–brachial pressure index over 12 months improved compared to baseline (*P* value 0.0001), **D** Total walking distance over 12 months improved compared to baseline (*P* value 0.0068), **E** Ulcer size over 12 months improved compared to baseline (*P* value < 0.0001)

### Rest pain score

Rest pain scores showed gradual and sustained decrease over the study period of 12 months. The mean (SD) rest pain scores reduced from 8.0 (1.57) at baseline to 4.6 (2.17) at 1 month, 2.8 (1.87) at 3 months, 1.7 (2.22) at 6 months and 1.1 (1.80) at 12 months which were statistically significant (*p* < 0.0001). GEE results showed that there was significant decrease in the rest pain scores by 0.47 units per month over a period of 12 months (p < 0.0001).

### Ulcer healing status

**Fig. 4:**
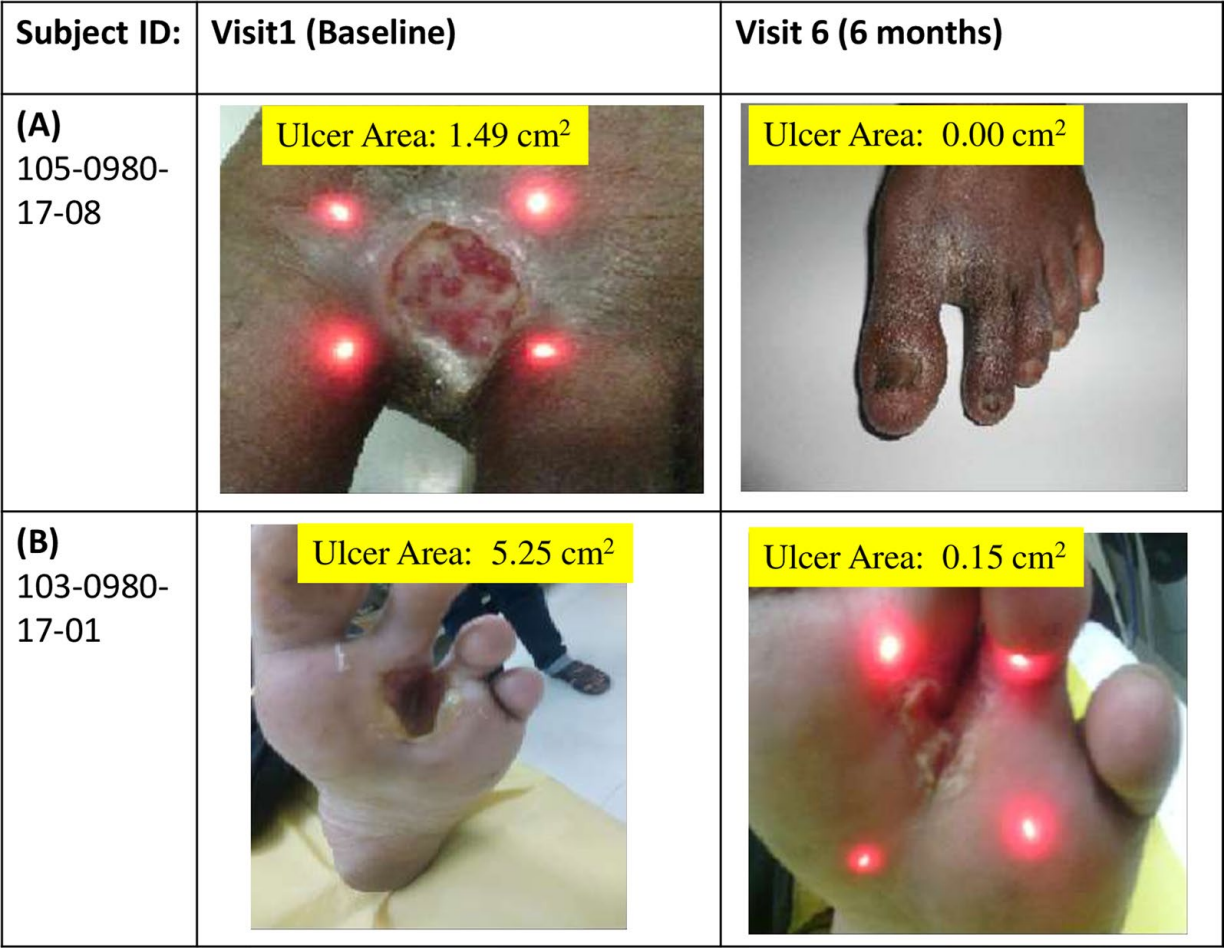
Ulcer healing status at different time points in two patients **A** Complete healing; **B** Partial healing

Out of 28 ulcers at baseline measured with the total surface area (cm^2^), 2 ulcers (7.1%) had complete healing and 16 ulcers (57.1%) had partial healing at 1 month. At 3 months, 10 ulcers (35.7%) had complete healing and 14 ulcers (50.0%) had partial healing. At 6 months, 17 ulcers (60.7%) had complete healing and 9 ulcers (32.1%) had partial healing, whereas 23 ulcers (82.1%) had complete healing and 4 ulcers (14.3%) had partial healing at 12 months. The statistically significant reduction in ulcer rate was observed as compared to baseline visit (*p* < 0.0001). No new ulcer was observed in any of the patient during the period of 12 months. Representative images of healing of ulcers are shown in Fig. 4.

### Ulcer size

The mean (SD) ulcer size (cm^2^) reduced from 3.98 (2.524) at baseline to 2.06 (2.104) at 1 month, 1.13 (1.509) at 3 months, 0.64 (2.126) at 6 months and 0.46 (2.124) at 12 months which were statistically significant. There was a significant increase in the ulcer rate of healing by 0.13 units per month for period of 12 months as per GEE analysis (*p* < 0.0001).

### ASP

Ankle systolic pressure (ASP) measured by Doppler is the higher of systolic pressure at posterior tibial or anterior tibial artery of the affected limb. The mean (SD) ASP increased from 61 (22.1) mmHg at baseline to 81 (22.4) mmHg at 1 month, 89 (22.1) mmHg at 3 months, 94 (25.3) mmHg at 6 months and 95 (27.6) mmHg at 12 months which were statistically significant (*p* < 0.0001). Longitudinal analysis of ASP by GEE method showed that there was a significant increasing pattern by 4.63 units per month over the period of 12 months compared to baseline (p < 0.0001).

### ABPI

Ankle–brachial pressure index (ABPI) is the ratio of ankle pressure (higher reading from the posterior tibial or anterior tibial artery of affected limb) to brachial pressure (higher of the two brachial readings).

The ABPI showed an increase over the study period of 12 months. The mean (SD) ABPI increased from 0.47 (0.156) at baseline to 0.61 (0.164) at 1 month, 0.67 (0.134) at 3 months, 0.70 (0.156) at 6 months and 0.73 (0.201) at 12 months which were statistically significant (*p* < 0.0001). GEE analysis showed that there was a statistically significant increase (*p* < 0.0001) by 0.02 units per month for 12 months.

### TWD

For calculation of total walking distance (TWD), patients were made to walk on a treadmill at 2 mph (3.2 km/h) up a 12% grade until they were forced to stop because of claudication or till maximum walking time of 60 min, whichever is earlier. Thereafter, TWD was calculated using the formula: Total Walking Distance = Total Walking time [Min] X 3.2 [Km/Hr]/60 [Min].

TWD showed an increase over the study period of 12 months. The mean (SD) TWD increased from 0.22 (0.254) at baseline to 0.29 (0.218) at 1 month, 0.44 (0.319) at 3 months, 0.64 (0.555) at 6 months and 0.88 (1.104) at 12 months which were statistically significant (*p* < 0.0001). GEE analysis showed that there was a statistically significant increase in the TWD by 0.06 units per month for 12 months (*p* < 0.0001).

#### Quality of Life (QoL)

Total score for QoL showed an increase over the study period of 12 months. The mean (SD) total score for quality of life increased from 2.39 (0.712) at baseline to 3.77 (1.315) at 1 month, 4.40 (0.873) at 3 months, 4.92 (1.185) at 6 months and 5.63 (1.044) at 12 months which were statistically significant (*p* < 0.0001). The change in efficacy parameters during the study duration is depicted in Tables 3 and 4.

**Table 3:** Mean and percentage change from baseline in RPS, ulcer size, ASP, ABPI, TWD and QoL

	Month 1/ Visit 4	Month 3/Visit 5	Month 6/Visit 6	Month 12/Visit 7
Rest Pain Score				
Absolute Mean (SD) Change from baseline	3.3 (2.11)	5.2 (2.19)	6.3 (2.72)	6.9 (2.53)
Percentage improvement from baseline	43.9%	64.5%	78.1%	84.9%
*P* value	< 0.0001	< 0.0001	< 0.0001	< 0.0001
Ulcer Size (cm^2^)				
Absolute Mean (SD) Change from baseline	1.92 (1.933)	2.85 (2.301)	3.34 (3.223)	3.52 (3.211)
Percentage improvement from baseline	48.41%	63.17%	79.68%	88.57%
* P* value	0.0416	< 0.0001	< 0.0001	< 0.0001
ASP (mmHg)				
Absolute Mean (SD) Change from baseline	20 (31.9)	27 (29.1)	32 (35.1)	34 (31.9)
Percentage improvement from baseline	17%	31%	37%	46%
* P* value	0.0058	0.0001	0.0002	< 0.0001
ABPI				
Absolute Mean (SD) increase from baseline	0.15 (0.226)	0.21 (0.200)	0.24 (0.209)	0.26 (0.211)
Percentage increase from baseline	17.76%	30.61%	37.06%	44.40%
	0.0042	< 0.0001	< 0.0001	< 0.0001
TWD (m/hr)				
Absolute Mean (SD) increase from baseline	0.07 (0.132)	0.23 (0.283)	0.42 (0.458)	0.67 (1.100)
Percentage improvement from baseline	47.10%	200.68%	268.72%	827.97%
* P* value	0.0123	0.0007	0.0002	0.0068
QoL				
Absolute Mean (SD) Change from baseline	1.39 (1.206)	2.01 (0.905)	2.53 (1.321)	3.24 (1.144)
Percentage improvement from baseline	66.31%	97.91%	124.95%	155.84%
* P* value	< 0.0001	< 0.0001	< 0.0001	< 0.0001

RPS: Rest Pain Score; ASP: Ankle Systolic Pressure; ABPI: Ankle–Brachial Pressure Index; TWD: Total Walking Distance; and QoL: Quality of Life

**Table 4:** Change in efficacy parameters at 12 months compared to baseline of the subjects participated in the study

Patient ID	Fontaine staging	Rest pain score			Ulcer size in cm^2^			TWD in km			ABPI		
		Baseline	After 6 months	After 12 months	Baseline	After 6 months	After 12 months	Baseline	After 6 months	After 12 months	Baseline	After 6 months	After 12 months
S001	IV	7.1	3.5	3.3	5.25	0.15	0	0	0.16	0.27	0.51	0.87	1.18
S002	IV	7.9	1.9	1.6	1.3	0	0	0.16	0.96	1.07	0.54	0.48	0.4
S003	IV	5.5	1.3	1	5.42	0	0	0.11	0.11	0.16	0.49	0.66	0.7
S004	IV	6.1	2.5	1.8	3.18	1.35	0.71	0.27	0.96	1.87	0.42	0.31	0.39
S005	IV	8.1	5.8	–	4.85	11.26	–	0.11	0.43	–	0.51	0.52	–
S006	IV	8.9	5.6	0	2.58	1.62	0	0.11	0.21	0.96	0.58	0.7	0.81
S007	IV	5.8	2	1	6.86	0.3	0.17	0.16	0.32	0.16	0.53	0.7	0.78
S008	IV	8.4	1.6	4.8	1.42	0.61	0.62	0.05	0.69	5.07	0	0.79	0.53
S009	IV	9.3	1.2	0	1.07	0	0	0	0.48	0.53	0.57	0.9	0.98
S010	IV	8.1	0	0	1.63	0	0	0.32	1.33	1.28	0.52	0.96	0.98
S011	IV	3.2	1.3	1.1	0.62	0.96	0.12	1.07	1.28	0.26	0.54	0.61	0.51
S012	IV	8.7	–	–	1.82	–	–	0.21	–	–	0.43	–	–
S013	IV	8.4	0	0	7.67	0.36	0	0	0.32	0.16	0.5	0.76	0.74
S014	IV	9.2	0	0	3.87	0	0	0.64	0.85	0.05	0.56	0.68	0.7
S015	IV	8.6	1.2	0	5.14	0.22	0	0	0	–	0.53	0.79	0.89
S016	IV	8.9	8.8	5.1	9.06	0	0	0.11	–	–	0.55	0.74	–
S017	IV	6.5	0	–	1.41	0	–	0.48	2.4	–	0	0.7	–
S018	IV	8.3	0.5	–	4.36	0	–	0.16	0.48	–	0.36	0.6	–
S019	IV	8.2	0	0	0.93	0	0	0.27	1.44	–	0.57	0.8	–
S020	IV	10	1	0	7.87	0	0	0	0.32	0.32	0.58	0.79	0.96
S021	IV	8.7	0	0	1.27	0	0	0.53	0.64	0.16	0.48	0.75	0.75
S022	IV	8.5	0.7	0.3	–	4.41	0	0.16	0.27	–	0.59	0.79	–
S023	IV	10	1	0	7.28	0.68	0	0	–	–	0.4	0.52	0.54
S024	IV	8.9	0	0	1.49	0	0	0.37	0.59	0	0.48	0.94	0.95

Rest Pain score measured with the help of visual analogue scale; TWD: total walking distance; and ABPI: ankle–brachial pressure index

Of the total of 24 patients dosed, 22 patients completed the study

Patient No. S012 was lost to follow-up after completion of Visit 5. Patient No. S005 discontinued from the study after completion of Visit 6

For subjects S016, S017, S018, S019 and S022 virtual visit (telephonic) was performed for Visit 7 (1 year)

Neither death nor life-threatening AEs were observed during the 12-month follow-up after stempeucel® therapy. Two serious AEs (necrotizing fasciitis and peripheral ischemia) were observed which were unrelated to the study drug. A total of 8 TEAEs were reported by 6 (25%) out of 24 patients in the study. Majority of the AEs were mild (4 events), 2 events were moderate, and 2 events were severe. All the AEs were evaluated and considered as unrelated to the study drug as the adverse experience being an expected symptom of disease under study or expected outcome of a previously existing or concurrent disease or concomitant medication or procedure as per the treating physician.

None of the patients underwent major amputation during the study; however, one patient required minor amputation at 6 months. There were no clinically significant abnormalities during laboratory assessments, physical examination and vital signs recordings during the study.

## Discussion

Patients with PAD require aggressive modification of risk factor, exercise rehabilitation, treatment with antiplatelet drugs, vasodilators, reconstruction of the blood circulation by endovascular interventions and bypass therapy [18, 19]. After ineffective attempts of revascularization, PAD patients undergo greater amputations of which only 25–50% achieve full mobility and the re-amputation rate is seen in 30% [20].

Various phases of randomized clinical trials have shown that cell therapy is a feasible option for CLI patients, using either intramuscular or intra-arterial (or combination of both) administration either by single or repeated administrations [16, 17, 21–26]. Comparison of these both routes of administration has shown similar outcomes [27, 28]. These studies have shown that the cells are both safe and efficacious by both the routes. Different cell types have been used in these trials, which includes CD133 + / CD34 + (cluster of differentiation) cells, unfractionated bone marrow concentrate, BM-MNCs (bone marrow-derived mononuclear stem cells), PB-MNCs (peripheral blood mononuclear cells) and bone marrow mesenchymal stromal cells (MSCs). The data suggested difference in therapeutic effect in various cell types (significant improvement to no improvement), and this may be related to the dose of the cells and the cell potency. Hence, a correct dose and assessment of potency marker, an important element for standardization of the cell product, may be important for consistent efficacy.

In the current study, stem cell was injected intramuscularly into the gastrocnemius muscle which might have created local depots of stem cells in the ischemic muscle, which will increase neovascularization by cell-to-cell contact, cell trans-differentiation and paracrine activity in the ischemic area. The angiogenic effect of the cells may be related to their ability to induce vascular and muscular regeneration by paracrine factors secreted. The angiogenic factors secreted by these cells especially vascular endothelial growth factor (VEGF) likely contribute to the increase in blood flow as evidenced by increase in ASP and ABPI in this study. Further, animal studies have also shown that intramuscular cell therapy may contribute to regeneration by tissue integration and/or secretion of paracrine factors [29, 30].

In this present study, multiple intramuscular injections of stempeucel® showed statistical significant reduction of rest pain and ulcer size along with healing of ulcers in an accelerated fashion as compared to baseline. There was statistically significant increased blood flow to the ischemic limbs as evidenced by increased ABPI and ASP. The QoL of patients improved along with increase in TWD. We have included no-option patients (patients without revascularization options) in the study which has a high rate of limb loss and death. In this study, no major amputation and no mortality were seen in the one-year follow-up period (amputation-free survival) and this may be due to increased neovascularization due to paracrine factors which has rescued the tissues from critical ischemia.

The mean time taken for complete epithelialization in our study was 435 days, while partial epithelization was observed within 375 days. Overall, the data suggest that more than 96% ulcers showed healing, which include more than 82% ulcers with complete healing. Such improvement in ulcer healing was also observed by Ponemone V et al. [31] and Hu MS et al.[32] The review article has documented that the MSCs promote cell migration, angiogenesis, epithelialization and granulation tissue formation, which result in accelerated wound closure. Moreover, Xie B et al. performed meta-analysis on randomized controlled clinical trials of CLI to assess the efficacy and safety of human autologous stem cell therapy, including bone marrow-derived MSCs (BM MSCs) in CLI. They too found that the cell therapy significantly increased the probability of ulcer healing [33].

Several preclinical and clinical studies have shown that administration of allogeneic MSCs in an unrelated mismatched allogeneic host does not stimulate the formation of allo-specific antibodies or lead to a T cell sensitization of the recipient to alloantigen in different animal models.^15^ Till date the safety of MSC-based therapy has been well established. Human clinical studies have shown no evidence of toxicity in terms of either aberrant differentiation or tumorogenicity. In our completed phase I/II study of CLI, we have shown that administration of stempeucel® did not adversely alter the immunological profile as it did not elicit T cells proliferative response in vivo and pro-inflammatory cytokines levels were comparable in both the cell and placebo arms at various time points[13]. In this study, none of the patients developed procedure-related complications and no death were observed during the one-year follow-up. All AEs and SAEs (serious adverse effects) were not related to the stempeucel® and were due to progress of the disease [34].

## Conclusion

MSCs are being implemented as a therapeutic option for the treatment of complications resulting from atherosclerotic PAD. The angiogenic cell therapy using MSCs in atherosclerotic PAD patients in our study has shown long-term improvement in limb ischemia, leading to extension of amputation-free survival, improved quality of life, improvement in rest pain score, ABPI and ASP and healing of ulcers. Stempeucel® in a dose of 2 million cells/kg body weight administered intramuscularly in the calf muscle and injected locally around the ulcers is a feasible option to treat patients suffering from CLI due to atherosclerotic PAD as shown by this label extension study.

## Data Availability

The datasets used and/or analyzed during the current study are available from the corresponding author on reasonable request.

## Abbreviations

ABPI: Ankle–brachial pressure index
AE: Adverse effects
ASP: Ankle systolic pressure
BD: Buerger’s disease
BM MNC: Bone marrow-derived mononuclear stem cells
BM MSC: Bone marrow-derived mesenchymal stromal cells
CD: Cluster of differentiation
CLI: Critical limb ischemia
CMV: Cytomegalovirus
CTRI: Clinical trials registry—India
GEE: Generalized estimating equation
HCV: Hepatitis C virus
HIV: Human immunodeficiency virus
ICH-GCP: International Council for Harmonization Good Clinical Practice
ICMR: Indian Council of Medical Research
mITT: Modified intention to treat
MRA: Magnetic resonance angiography
PAD: Peripheral arterial disease
PB MNC: Peripheral blood mononuclear cells
QoL: Quality of life
SAE: Serious adverse effect
SD: Standard deviation
SDF: Stromal cell-derived factor
TEAE: Treatment emergent adverse effects
TPHA: Treponema pallidum hemagglutination assay
TWD: Total walking distance
VEGF: Vascular endothelial growth factor

## References

[1] Bosevski M (2012). Peripheral arterial disease and diabetes. Pril.

[2] Suzuki J, Shimamura M, Suda H, Wakayama K, Kumagai H, Ikeda Y (2016). Current therapies and investigational drugs for peripheral arterial disease. Hypertens Res.

[3] Sprengers RW, Lips DJ, Moll FL, Verhaar MC (2008). Progenitor cell therapy in patients with critical limb ischemia without surgical options. Ann Surg.

[4] Aday AW, Matsushita K (2021). Epidemiology of peripheral artery disease and polyvascular disease. Circ Res.

[5] Shu J, Santulli G (2018). Update on peripheral artery disease: epidemiology and evidence-based facts. Atherosclerosis.

[6] Dormandy J, Heeck L, Vig S (1999). The fate of patients with critical leg ischemia. Semin Vasc Surg.

[7] Kim HO, Kim W (2018). Elucidation of the diagnosis and treatment of peripheral arterial disease. Korean Circ J.

[8] Zemaitis MR, Boll JM, Dreyer MA. Peripheral Arterial Disease. StatPearls. 2022. 28613496

[9] Tachi Y, Fukui D, Wada Y, Koshikawa M, Shimodaira S, Ikeda U (2008). Changes in angiogenesis-related factors in serum following autologous bone marrow cell implantation for severe limb ischemia. Expert Opin Biol Ther.

[10] Amann B, Luedemann C, Ratei R, Schmidt-Lucke JA (2009). Autologous bone marrow cell transplantation increases leg perfusion and reduces amputations in patients with advanced critical limb ischemia due to peripheral artery disease. Cell Transplant.

[11] Molavi B, Zafarghandi MR, Aminizadeh E, Hosseini SE, Mirzayi H, Arab L (2016). Safety and efficacy of repeated bone marrow mononuclear cell therapy in patients with critical limb ischemia in a pilot randomized controlled trial. Arch Iran Med.

[12] Liang TW, Jester A, Motaganahalli RL, Wilson MG, G'Sell P, Akingba GA (2016). Autologous bone marrow mononuclear cell therapy for critical limb ischemia is effective and durable. J Vasc Surg.

[13] Gupta PK, Krishna M, Chullikana A, Desai S, Murugesan R, Dutta S (2017). Administration of adult human bone marrow-derived, cultured, pooled, allogeneic mesenchymal stromal cells in critical limb ischemia due to buerger’s disease: phase ii study report suggests clinical efficacy. Stem Cells Transl Med.

[14] Asahara T, Masuda H, Takahashi T, Kalka C, Pastore C, Silver M (1999). Bone marrow origin of endothelial progenitor cells responsible for postnatal vasculogenesis in physiological and pathological neovascularization. Circ Res.

[15] Rasmusson I (2006). Immune modulation by mesenchymal stem cells. Exp Cell Res.

[16] Gupta PK, Chullikana A, Parakh R, Desai S, Das A, Gottipamula S (2013). A double blind randomized placebo controlled phase I/II study assessing the safety and efficacy of allogeneic bone marrow derived mesenchymal stem cell in critical limb ischemia. J Transl Med.

[17] Gupta PK, Dutta S, Kala S, Nekkanti M, Desai SC, Mahapatra SS (2021). Phase IV postmarketing surveillance study shows continued efficacy and safety of Stempeucel in patients with critical limb ischemia due to Buerger’s disease. Stem Cells Transl Med.

[18] Biancari F (2013). Meta-analysis of the prevalence, incidence and natural history of critical limb ischemia. J Cardiovasc Surg.

[19] Nehler MR, Duval S, Diao L, Annex BH, Hiatt WR, Rogers K (2014). Epidemiology of peripheral arterial disease and critical limb ischemia in an insured national population. J Vasc Surg.

[20] Sprengers RW, Lips DJ, Bemelman M, Verhaar MC (2007). Lower leg amputation due to critical limb ischaemia: morbidity, mortality and rehabilitation potential. Ned Tijdschr Geneeskd.

[21] Barć P, Skóra J, Pupka A (2006). Bone-marrow cells in therapy of critical limb ischemia of lower extremities - own experience. Acta Angiol..

[22] Iafrati MD, Hallett JW, Geils G, Pearl G, Lumsden A, Peden E (2011). Early results and lessons learned from a multicenter, randomized, double-blind trial of bone marrow aspirate concentrate in critical limb ischemia. J Vasc Surg.

[23] Lu D, Chen B, Liang Z, Deng W, Jiang Y, Li S (2011). Comparison of bone marrow mesenchymal stem cells with bone marrow-derived mononuclear cells for treatment of diabetic critical limb ischemia and foot ulcer: a double-blind, randomized, controlled trial. Diabetes Res Clin Pract.

[24] Walter DH, Krankenberg H, Balzer JO, Kalka C, Baumgartner I, Schlüter M (2011). Intraarterial administration of bone marrow mononuclear cells in patients with critical limb ischemia a randomized-start, placebo-controlled pilot trial (PROVASA). Circ Cardiovasc Interv.

[25] Ozturk A, Kucukardali Y, Tangi F, Erikci A, Uzun G, Bashekim C (2012). Therapeutical potential of autologous peripheral blood mononuclear cell transplantation in patients with type 2 diabetic critical limb ischemia. J Diabetes Complicat.

[26] Powell RJ, Marston WA, Berceli SA, Guzman R, Henry TD, Longcore AT (2012). Cellular therapy with ixmyelocel-T to treat critical limb ischemia: the randomized, double-blind, placebo-controlled RESTORE-CLI trial. Mol Ther.

[27] Gu YQ, Zhang J, Guo LR, Qi LX, Zhang SW, Xu J (2008). Transplantation of autologous bone marrow mononuclear cells for patients with lower limb ischemia. Chin Med J.

[28] Klepanec A, Mistrik M, Altaner C, Valachovicova M, Olejarova I, Slysko R (2012). No difference in intra-arterial and intramuscular delivery of autologous bone marrow cells in patients with advanced critical limb ischemia. Cell Transplant.

[29] Sasaki S, Inoguchi T, Muta K, Abe Y, Zhang M, Hiasa K (2007). Therapeutic angiogenesis by ex vivo expanded erythroid progenitor cells. Am J Physiol Hear Circ Physiol.

[30] Sasaki K, Heeschen C, Aicher A, Ziebart T, Honold J, Urbich C (2006). Ex vivo pretreatment of bone marrow mononuclear cells with endothelial NO synthase enhancer AVE9488 enhances their functional activity for cell therapy. Proc Natl Acad Sci USA.

[31] Ponemone V, Gupta S, Sethi D, Suthar M, Sharma M, Powell RJ (2017). Safety and effectiveness of bone marrow cell concentrate in the treatment of chronic critical limb ischemia utilizing a rapid point-of-care system. Stem Cells Int.

[32] Hu MS, Borrelli MR, Lorenz HP, Longaker MT, Wan DC (2018). Mesenchymal stromal cells and cutaneous wound healing: a comprehensive review of the background, role, and therapeutic potential. Stem Cells Int.

[33] Xie B, Luo H, Zhang Y, Wang Q, Zhou C, Xu D (2018). Autologous stem cell therapy in critical limb ischemia: a meta-analysis of randomized controlled trials. Stem Cells Int..

[34] Liew A, O’Brien T (2012). Therapeutic potential for mesenchymal stem cell transplantation in critical limb ischemia. Stem Cell Res Ther.

